# Rescue of cognitive deficits in APP/PS1 mice by accelerating the aggregation of β-amyloid peptide

**DOI:** 10.1186/s13195-019-0560-6

**Published:** 2019-12-17

**Authors:** Jian-Xiang Zhang, Yi-Hui Lai, Pan-Ying Mi, Xue-Ling Dai, Ran Zhang, Zhan-Jun Zhang, Shu-Juan Zhang, Xi-Wen Zhang, Xi-Yan Zhang, Bing-Yu Yang, Dong-Mei Cui, Chen Zhang, Chang-Qi Zhao, Fei Dou

**Affiliations:** 1 Beijing Key Laboratory of Genetic Engineering Drugs and Biotechnology, College of Life Sciences, Beijing Normal University, Beijing, 100875 China; 2 Beijing Key Laboratory of Bioactive Substances and Functional Foods, Beijing Union University, Beijing, 100191 China; 3 State Key Laboratory of Cognitive Neuroscience and Learning, Beijing Normal University, Beijing, 100875 China; 4 Peking University China-Japan Friendship School of Clinical Medicine, Beijing, 100029 China; 5 No. 161 Middle School, Beijing, 100006 China; 6 College of Pharmaceutical Sciences, Zhejiang University of Technology, Hangzhou 310014 Zhejiang, People’s Republic of China; 7 College of Pharmaceutical Sciences, Zhejiang University, Hangzhou 310058 Zhejiang, People’s Republic of China

**Keywords:** Alzheimer’s disease, β-Amyloid, Oligomers, Globulomers, Flavonoid

## Abstract

**Background:**

Brain amyloid deposition is one of the main pathological characteristics of Alzheimer’s disease (AD). Soluble oligomers formed during the process that causes β-amyloid (Aβ) to aggregate into plaques are considered to have major neurotoxicity. Currently, drug development for the treatment of Alzheimer’s disease has encountered serious difficulties. Our newly proposed solution is to accelerate the aggregation of Aβ to reduce the amount of cytotoxic Aβ oligomers in brain tissue. This strategy differs from the existing strategy of reducing the total Aβ content and the number of amyloid plaques.

**Method:**

In this study, we screened a small library and found that a flavonoid compound (ZGM1) promoted the aggregation of β-amyloid (Aβ). We further verified the binding of ZGM1 to Aβ42 using a microscale thermophoresis (MST) assay. Subsequently, we used dot blotting (DB), transmission electron microscopy (TEM), and thioflavin T fluorescence (ThT) measurements to study the aggregation of Aβ under the influence of ZGM1. By using cell experiments, we determined whether ZGM1 can inhibit the cytotoxicity of Aβ. Finally, we studied the protective effects of ZGM1 on cognitive function in APPswe/PS1 mice via behavioral experiments and measured the number of plaques in the mouse brain by thioflavin staining.

**Results:**

ZGM1 can bind with Aβ directly and mediate a new Aβ assembly process to form reticular aggregates and reduce the amount of Aβ oligomers. Animal experiments showed that ZGM1 can significantly improve cognitive dysfunction and that Aβ plaque deposition in the brain tissue of mice in the drug-administered group was significantly increased.

**Conclusion:**

Our research suggests that promoting Aβ aggregation is a promising treatment method for AD and deserves further investigation.

## Background

Amyloid plaque accumulation is one of the two pathological features of Alzheimer’s disease (AD). These plaques are mainly formed by β-amyloid peptide (Aβ), which consists of 39–42 amino acid residues and is produced by the amyloidogenic pathway via the hydrolysis of amyloid precursor protein (APP) by β-secretase and γ-secretase [1, 2]. Since the discovery of the involvement of APP gene mutants in familial AD in 1991, the amyloid cascade hypothesis has gradually become the mainstream theory of the pathogenesis of AD, and the discovery of presenilin 1 (PS1) protein mutants has further reinforced this hypothesis [3]. Researchers have put much effort into the treatment of AD by reducing Aβ levels or inhibiting the formation of Aβ plaques by using drugs that block Aβ production by inhibiting γ-secretase or β-secretase or that reduce the aggregation of Aβ monomers, and immunological attempt strategies have also been attempted by using anti-Aβ antibodies. However, although these methods were effective in preventing the production and aggregation of Aβ and even succeeded in animal experiments, they showed poor efficacy or significant side effects in clinical trials. Therefore, it is crucial to find new methods to treat Alzheimer’s disease [4].

The aggregation of Aβ involves a complex nucleation-dependent polymerization process with multiple stages and intermediates [5]. Accumulating evidence suggests that it is intermediates, such as Aβ soluble oligomers (AβOs), rather than mature amyloid fibrils that cause neuronal dysfunction and memory impairment in AD [6, 7].

The earliest evidence for the toxicity of AβOs came from fluid dynamics experiments, which found that AβOs in solution could not be separated by centrifugation [8]. The most frequently reported AβOs are endogenous Aβ dimers, trimers, and Aβ*56 isolated from the brain tissue of AD model mice or patients and soluble Aβ aggregates obtained in vitro, such as Aβ-derived diffusible ligands (ADDLs) [9]. AβOs usually have an intermolecular parallel β-sheet structure, but it has also been reported that some AβOs have a random coil-like structure, and these structures can convert from one to the other under specific conditions [10]. AβOs formed in different ways can produce similar cellular metabolic effects [11] and can be recognized by structurally specific antibodies [12]. In AD models, the emergence of AβOs coincides with the onset of memory dysfunction [13]. When injected into animal models, AβOs from AD brains and synthetic Aβ disrupt synaptic plasticity as well as learning and memory [14]. In addition, antibodies against AβOs restored memory function in AD models [6, 15].

In summary, because the complicated process of Aβ aggregation is not well understood, it is not clear which form of Aβ causes cell death and eventually leads to AD, so reducing the total Aβ amount in brain tissue has been the main principle used for screening drugs to treat AD. Recent studies have shown that soluble AβOs are a major cause of the loss of synaptic function and neurons compared to mature amyloid fibrils and Aβ monomers [6]. Therefore, we believe that lowering the levels of AβOs by small molecule drugs is a more reasonable therapeutic strategy than inhibiting the formation of Aβ fibrils. There are two strategies used for reducing the content of Aβ oligomers, one of which is to inhibit monomer aggregation into oligomers. However, recent studies have shown that Aβ dimers can disrupt the homeostasis of glutamate, leading to the abnormal activation and impairment of neurons [16]. This means that therapeutic drugs should interact with almost all Aβ monomers. In this case, the drug should work in a manner similar to the binding of detergent and hydrophobic protein, and the molar ratio of the compound to Aβ needs to be high to achieve this effect, which limits its feasibility. The other strategy is to accelerate the conversion of the monomer into polymers by skipping the stage of oligomer formation. We believe that the second strategy is a more feasible method.

Here, we discovered a small flavanol compound, 2-(2,3-dihydroxyphenyl)-5,7-dimethoxychroman-4-one (ZGM1), which promoted the aggregation of Aβ monomers and led to an assembly process that formed reticular aggregate, thereby reducing the amount of toxic Aβ oligomers. In addition, we proved the preventive effect of ZGM1 in AD mice, which provided a new strategy for the prevention and treatment of AD.

## Method

### In vitro studies

#### Synthesis of ZGM1

(2,3-Bis (methoxymethoxy)phenyl)-5,7-dimethoxychroman-4-one (0.2 mmol) was dissolved in 2 M HCl (10 mL), and then the mixture was stirred while refluxing for 2 h. The concentrated mixture was treated with cold water and extracted with ethyl acetate. The ethyl acetate layer was washed with brine, dried over anhydrous magnesium sulfate, and concentrated in vacuo. ZGM1 was finally produced from the residue that was purified on a silica gel column.

#### Preparation of Aβ aggregates

Synthetic Aβ42 peptides (Abcam, ab120301) or synthetic Aβ40 peptides (Abcam, ab120479) were dissolved in DMSO to obtain a 5 mM stock solution. Each stock solution was then diluted with D-PBS. ZGM1 was dissolved in DMSO to obtain a 0.1 M stock solution and then diluted with D-PBS to obtain the working concentration. Aβ was incubated with ZGM1 at 37 °C for 12–120 h at specific proportions. Congo red, which has been reported to inhibit Aβ aggregation, was used as a control.

#### Blotting analyses

A total of 3 μl of each sample was incubated for 0 h, 12 h, and 24 h with 3 μl Nu-PAGE™ LDS Sample Buffer (Invitrogen) and 6 μl ddH_2_O. The mixture was electrophoresed in a Nu-PAGE 4%~ 12% Bis-Tris premade gel (Invitrogen NP0321BOX) at 200 V for 30 min, and the protein was transferred to a 0.2 μm PVDF membrane. The 6E10 antibody was used for Western blotting to detect Aβ aggregation. Another 2 μl of the sample was incubated for 24–120 h or was not incubated and was spotted on an NC membrane (Sangon) for dot blotting analysis to detect Aβ oligomers.

#### Thioflavin T fluorescence

Thioflavin T (ThT, Sigma) was dissolved in DMSO to obtain a 0.1 M stock solution. The ThT, Aβ, and ZGM1 stock solutions were diluted with D-PBS. Aβ (30 μM) was centrifuged at 17,000×*g* for 20 min, and then the supernatant was retained for subsequent experiments. These reagents were mixed at a ratio of 1:1:1 so that the final concentration of Aβ was 10 μM. Then, the mixtures were added to a black-walled 96-well plate and incubated at 37 °C, and the fluorescence signals were detected at 0 h, 28 h, 50 h, 72 h, 98 h, 118 h, and 166 h. The excitation wavelength was 440 nm, and the emission wavelength was 476.5 nm.

#### Transmission electron microscopy

The edge of the copper mesh was clamped with tweezers, and 6 μl of the incubated sample was added to the center of the front side of the copper mesh and allowed to remain for 90 s. The sample was gently removed with absorbent paper, and a drop of uranyl acetate was added to the front of the copper mesh and immediately removed. The processed was repeated. After the third drop of uranyl acetate was added, it was allowed to remain on the mesh for 30 s before being removed. The copper mesh was dried and put into the storage box for observation. The images were obtained by transmission electron microscopy (FEI Tecnai Spirit with iCorr D1319, Tsinghua University).

#### Microscale thermophoresis

Aβ42 linked to a 5-carboxyfluorescein tag at the N-terminus (5’FAM-Aβ42, Chinese Peptide) was dissolved in DMSO to obtain a 5 mM stock solution. Each stock solution was diluted with D-PBS to obtain a concentration of 400 nM and centrifuged at 17,000×*g* for 20 min at 4 °C, and then the supernatant was retained. The ZGM1 stock solution was diluted to a concentration of 2 mM with D-PBS. ZGM1 was titrated at a 1:1 dilution 16 times beginning at 2 mM. 5’FAM-Aβ was added to each tube and mixed; the final concentration of 5’FAM-Aβ was 200 nM, and the highest concentration of ZGM1 was 1 mM. A capillary tube (NanoTemper, MO-K002) was inserted into each tube to allow the sample to enter the capillary. The capillary was placed in each sample well in order of the ZGM1 concentration (from low to high) and was detected using microscale thermophoresis (MST, NanoTemper, Monolith NT.115).

#### Primary culture of cortical neurons

Mice at 17–18 days of pregnancy were sacrificed. The abdominal cavity was carefully opened, and the embryos were removed; the whole brain was also removed and placed in DMEM/F12 (1:1) medium. The olfactory bulb and brain stem were removed, and the vascular membrane was peeled off. The remaining tissue was crushed with a yellow pipet tip, transferred into a 15 mL centrifuge tube containing 0.05% Trypsin (Gibco, 25300054), placed on ice for 15 min, and then incubated at 37 °C for 10–15 min for digestion. Most of the supernatant was aspirated. Then, 50 μL DNase I (Thermo, EN0523) was added, and the tissue was digested at 37 °C for 3 min. A total of 10 mL of DMEM/F12 (1:1) medium containing 10% FBS was added to terminate digestion. The mixture was mixed 20 times by aspiration and then centrifuged at 1200 rpm for 4 min, and the supernatant was discarded. The cells were resuspended gently in 3 ml of neurobasal (Gibco, 21103049) medium, and then a cell sieve (40 μm, Falcon, 352340) was used to filter the sample. The cells were diluted in adherent medium (89.75% neurobasal medium + 10% FBS + 0.25% GlutaMAX) and then inoculated into 96-well plates at a density of 40,000 cells per well. The growth medium (97.75% neurobasal medium + 2% B27 + 0.25% GlutaMAX) was prewarmed for fluid exchange after 4 h.

#### Cell culture

SH-SY5Y cells were grown in 1:1 DMEM/F12 medium (Thermo, 11330032) supplemented with 10% fetal bovine serum (Biowest), 0.5% MEM non-essential amino acids 100× solution (NEAA, Thermo), and 0.5% sodium pyruvate 100× solution (SP, Thermo) at 37 °C with 5% CO_2_ in a humidified atmosphere.

#### CCK-8 assays

The experimental groups were treated with ZGM1, Aβ, or both, while the control groups were treated with an equal amount of DMSO; each gradient used three replicates. After 48 h, the medium was changed to a medium containing 5% CCK-8 reagent (BBI, E606335), and the incubation was continued for 1 h in the dark. Then, the absorbance was measured at a wavelength of 450 nm using a microplate reader (Polarstar Omega, BMG Labtech).

### Mouse feeding and intragastric administration

All animal experiments were carried out in accordance with the National Institutes of Health Guide for the Care and Use of Laboratory Animals (NIH Publications No. 8023, revised 1978) and Regulations for the Administration of Affairs Concerning Experimental Animals (China, Revised in 2011). APP/PS1 mice and 129/C57BL/6 wild type mice were purchased from the Model Animal Research Center of Nanjing University and were raised in the same environment with sufficient food and water and a light to dark cycle of 12:12 h.

Four-month-old APP/PS1 TG mice (male) were divided into three groups: Group T, L-Z, and H-Z, and 4-month-old WT (male) comprised the W group. The dry ZGM1 powder was resuspended in 0.1% sodium carboxymethylcellulose (SCC, Solarbio). The drug was delivered into the stomach by a gavage needle, and the dosage was determined by the body weight. The dosage of the L-D group was 40 mg/kg per day, and the dosage of the H-D group was 120 mg/kg per day. Groups W and T were fed an equal amount of 0.1% SCC as controls for 8 weeks. The mice were administered the drug daily except for the weekends.

### ZGM1 tissue abundance analysis

The mice were orally administered the drug at a dosage of 40 or 120 mg/kg. The mice were sacrificed by perfusion, and the brain tissue was obtained. The brain tissue, an equal mass of glass beads (Sigma, G8772), and 4 volumes of methanol prechilled at − 80 °C were added to the homogenate tubes, and the mixture was homogenized (6000 g) for 30 s and then cooled for 1 min on ice, which was repeated 6 times. The homogenate was placed at − 80 °C for 2 h. The homogenate was centrifuged at 15000×*g* for 15 min at 4 °C, and 200 μL of the supernatant was added to a new 1.5 mL EP tube. The 0 h mouse blood sample was used as a blank. ZGM1 powder was dissolved to generate a standard curve for the absolute quantification of ZGM1 in the samples. All samples and standards were tested by the Metabolomics Facility at the Technology Center for Protein Science, Tsinghua University.

### Behavioral studies

The data analyses, including the recordings of all behavioral responses, were transcribed manually into a computer-acceptable format by researchers blinded to the group assignments.

#### Open-field experiment

The movement and retention information of the mice over 5 min were recorded by a camera. The next mouse experiment was performed after cleaning the field with 75% alcohol. After all experiments were completed, the total distance, the central area, and the number of crossings in the central area were analyzed.

#### Elevated plus maze

After placing the mice in the center of the elevated plus maze (80 cm × 80 cm), the movement and retention information of the mice over 5 min were recorded by a camera located at the top of the room. The next mouse experiment was performed after cleaning the maze with 75% alcohol. After all experiments were finished, the open arm entry and time in the open arm were statistically analyzed, and the differences between the groups were compared.

#### Novel object recognition

On the first day, objects A (circles) and B (squares) of the same color were placed in the open field. The mouse was free to explore in this environment for 10 min. The movement and retention information of the mice were recorded. The next mouse experiment was performed after cleaning the field with 75% alcohol. The next day, object B was replaced in the open field by a circular object C, which was slightly larger than object A. The position of object A did not change. After the mice were placed in the field, the movement and retention information of the mice were recorded over 5 min. The number of times and the timing of the sniffing of objects A and C by the mice were statistically analyzed, and the differences between the groups were compared.

#### Morris water maze

A platform was placed 1.5 cm below the water surface in the second quadrant of a circular water tank (80 cm diameter, 25 ± 1 °C). Titanium dioxide was added to whiten the water. The test was performed for 6 days. Mice were trained for 6 consecutive days with 3 trials per day as acquisition trials. Each trial began with placing the mouse into a different quadrant and allowing it to swim freely for 60 s. After each mouse reached the platform (or were guided to the platform if the mice were unable to locate the platform after 60 s), they were returned to their cage to dry for 20 min. The time that each mouse took to reach the platform was regarded as the escape latency. On the 7th day, the mice were rested for a day. On the 8th day, a probe test was performed without the platform for 60 s. Each mouse was placed into the opposite quadrant of the target zone, from which the platform was removed. The time in the target zone and the number of crossings that occurred in the probe were recorded.

### ThS staining of Aβ plaques in brain sections

After the behavioral studies, the mice were deeply anesthetized with 7% chloral hydrate (dissolved in PBS, 0.02 mL/g, intraperitoneally) and perfused. For ThS staining, excised brains were fixed overnight in 4% paraformaldehyde at 4 °C and immersed in 30% sucrose for 24 h twice for dehydration. Brain slices were cut in the coronal plane (30 μm per section) at − 26 °C. Aβ plaques in brains were visualized using ThS staining. ThS was dissolved in 50% ethanol at 0.4 M (filtered by a 0.22-μm filter), and brain sections were stained for 8 min and then washed with 50% ethanol for 3 min, which was repeated 3 times. The slices were finally sealed with 20% glycerin (dissolved in PBS). The images of the plaques were obtained with an Olympus fluorescence microscope. The number and areas of the plaques were determined using the ImageJ program (NIH). The analyses of the plaque distributions were transcribed manually into a computer-acceptable format by researchers blinded to the group assignments.

### Blotting analyses and the quantification of Aβ in brain lysates

Brain tissues were homogenized in ice-cold RIPA containing a 1* proteinase inhibitor. The homogenized tissue was ultrasonically crushed on ice for 20 min at 5% of the maximum power. The supernatant of the brain lysate was used for ELISA of Aβ40 or Aβ42 and Western blotting and dot blotting to detect biochemical changes and soluble Aβ. The concentrations of the soluble fractions of brain lysates were determined by using a BCA protein assay kit (Thermo, A53225). Protein samples (20 μg) were loaded in each lane of SDS–PAGE gels for Western blot analyses. GluR1, GluR2, PSD95, synaptotagmin, and App695 were measured. The measurements of Aβ40 and Aβ42 content were completed with an ELISA kit (Invitrogen) according to the manufacturer’s instructions with fivefold diluted soluble fraction samples. Detailed information about the antibodies used is included in the Additional files.

### Statistical analysis

Graphs were obtained with GraphPad Prism 6, and statistical analyses were performed with one-way analysis of variance or two factors analysis of variance (*P < 0.05, **P < 0.01, ***P < 0.001, other comparisons were not significant).

The error bars represent the s.e.m. or s.d.

### Data availability

The data that support the findings of this study are available within the article, in the Additional files, and from the corresponding author upon reasonable request.

## Results

### ZGM1 promotes Aβ42 monomer aggregation

**Fig. 1:**
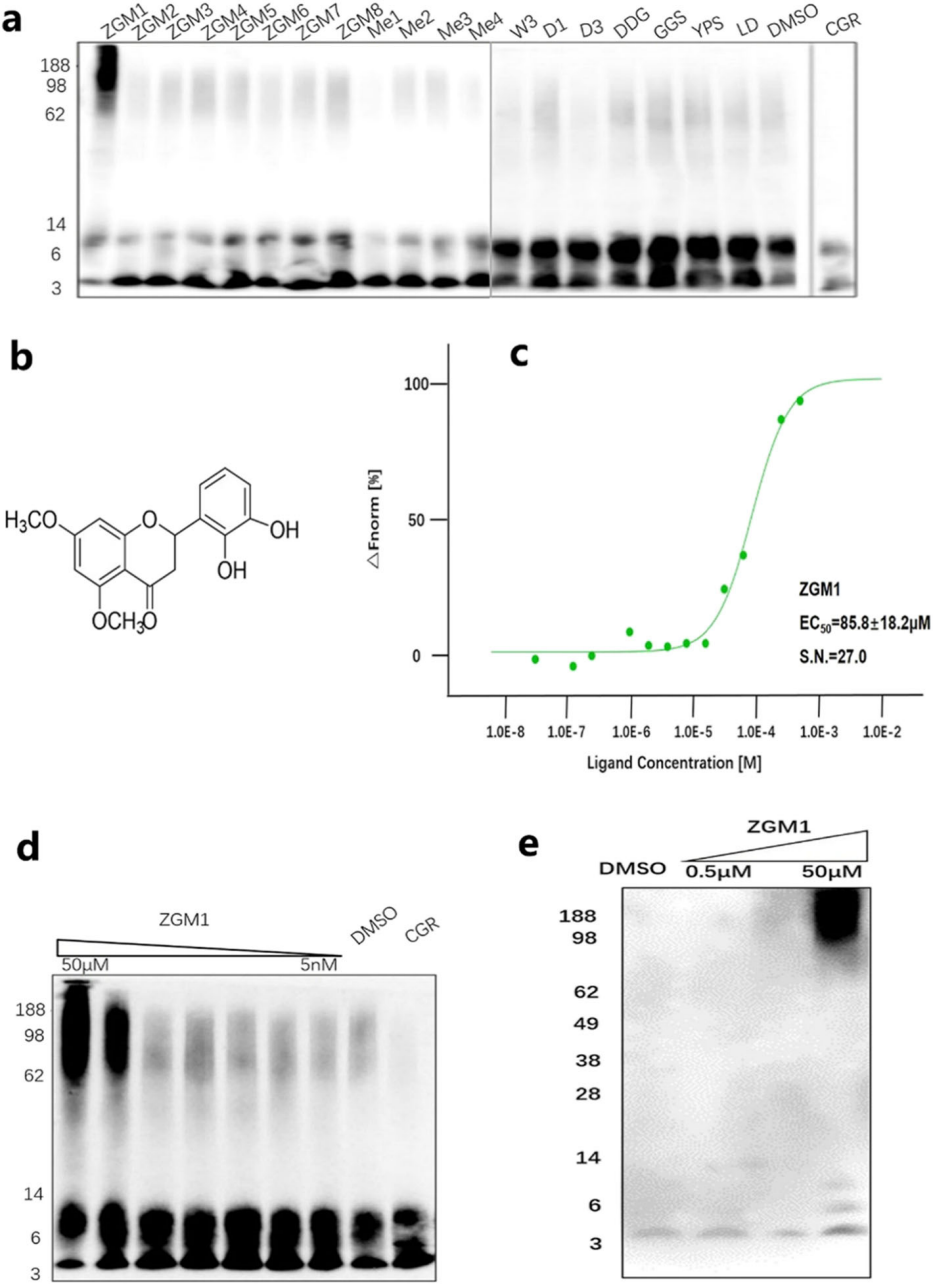
| ZGM1 binds to the Aβ monomer and promotes its aggregation. **a** Western blot analysis of Aβ42 (50 μM) aggregation after incubation with a series of small molecule compounds (50 μM) for 24 h; DMSO is the solvent; CGR refers to Congo red, which has been reported to inhibit Aβ aggregation; the Aβ-specific antibody 6E10 was used. **b** Structural formula of ZGM1. **c** MST assays. ZGM1 can directly bind to Aβ. EC50 = 85.8 ± 18.2 μM, S.N. = 27.0. **d** Western blot analysis of Aβ42 (50 μM) aggregates incubated with different concentrations of ZGM1 at 37 °C for 24 h. Lane 9: control (CGR) with Congo red, which inhibits Aβ aggregation; lane 8: control (DMSO) with the same content of DMSO as the other samples but without ZGM1. Lanes 2–7: Aβ42 (50 μM) treated with 50 μM, 5 μM, 1 μM, 200 nM, 50 nM, and 5 nM ZGM1. **e** Western blot analysis of Aβ40 (50 μM) aggregates incubated with different concentrations of ZGM1 at 37 °C for 24 h. Lane 1: control treated with the same content of DMSO as the other samples but without ZGM1; lane 2–4: Aβ40 (50 μM) treated with 0.5 μM, 5 μM, and 50 mM ZGM1

We screened a small flavonoid compound library (ZGM series, see Table 1; NMR data, see Additional file 1: Table S1) to identify compounds that could accelerate the aggregation of Aβ42 into high molecular weight aggregates (Fig. 1a). To achieve this goal, we performed Western blotting experiments using the Aβ-specific antibody 6E10 to detect the degree of the aggregation of Aβ42 after adding different compounds. We found that the flavonoid 2-(2,3-dihydroxyphenyl)-5,7-dimethoxychroman-4-one (ZGM1, Fig. 1b, Additional file 1: Figure S1), which is synthesized from gallic acid and 2,3-dimethoxybenzaldehyde, could significantly accelerate the aggregation of Aβ42 (Fig. 1b).

**Table 1:**
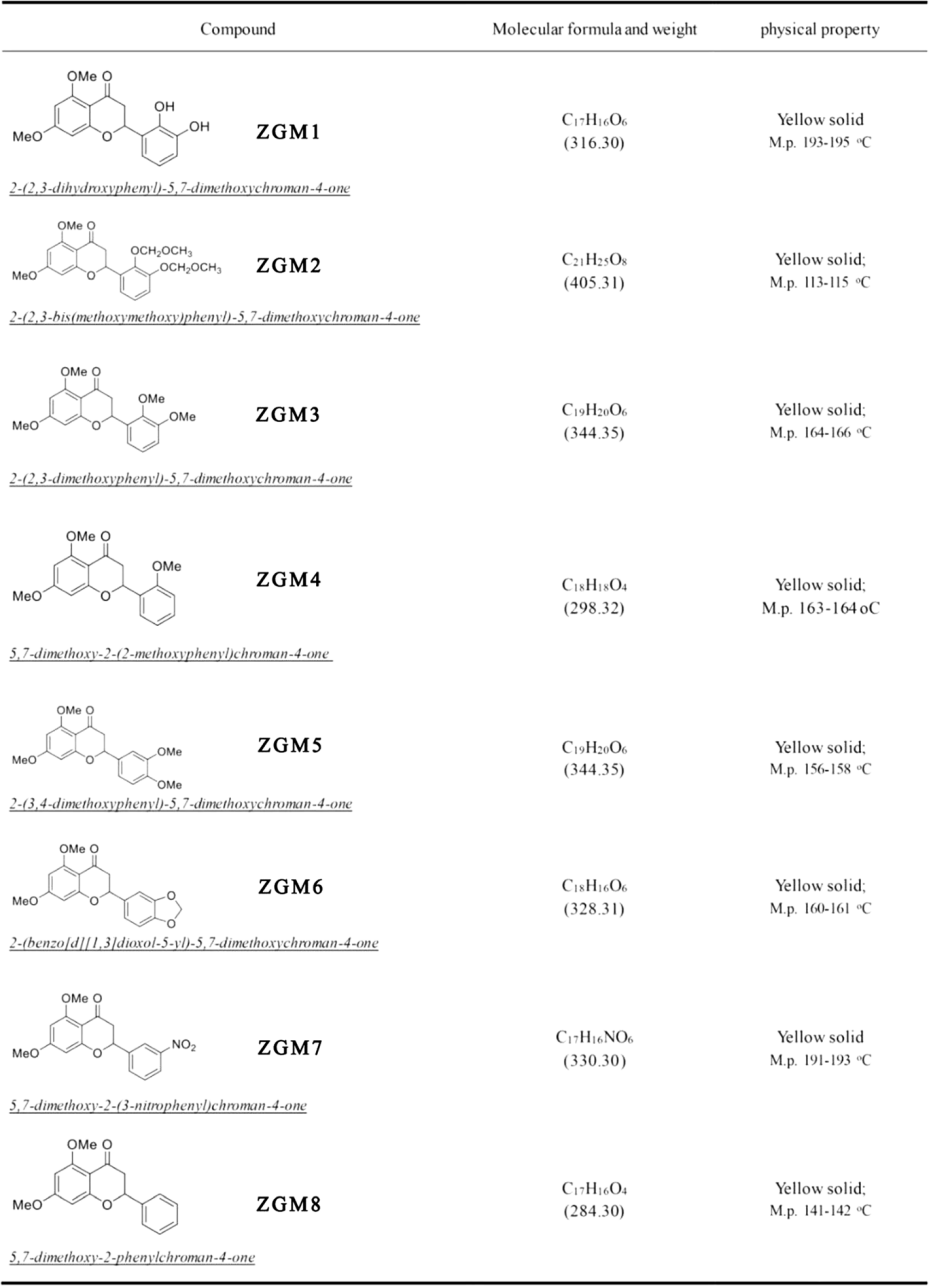
List of all tested ZGM compounds

### ZGM1 binds to Aβ monomers and dose-dependently promotes the aggregation of Aβ monomers

To confirm the interaction between the ZGM1 and Aβ42 monomers, we used 5’FAM N-end labeled Aβ42 (5’FAM-Aβ42) to verify binding by using microscale thermophoresis (MST). The quantitative analysis of the data revealed that the half-maximal binding (EC50) value was 85.8 ± 18.2 μM (Fig. 1c). Furthermore, we mixed 50 μM Aβ42 with different concentration gradients of ZGM1 and incubated it at 37 °C for 48 h, after which we detected the degree of the aggregation of Aβ42 in the different groups by Western blotting. ZGM1 showed a dose-dependent effect on the aggregation of Aβ42 monomers and showed significant enhancement when the molar ratio of ZGM1 to Aβ42 was 1:10 (Fig. 1d). Furthermore, ZGM1 was also effective in promoting the aggregation of Aβ40, which is more difficult to aggregate (Fig. 1e).

### Different concentrations of ZGM1 have different effects on Aβ amyloid fibril formation

**Fig. 2:**
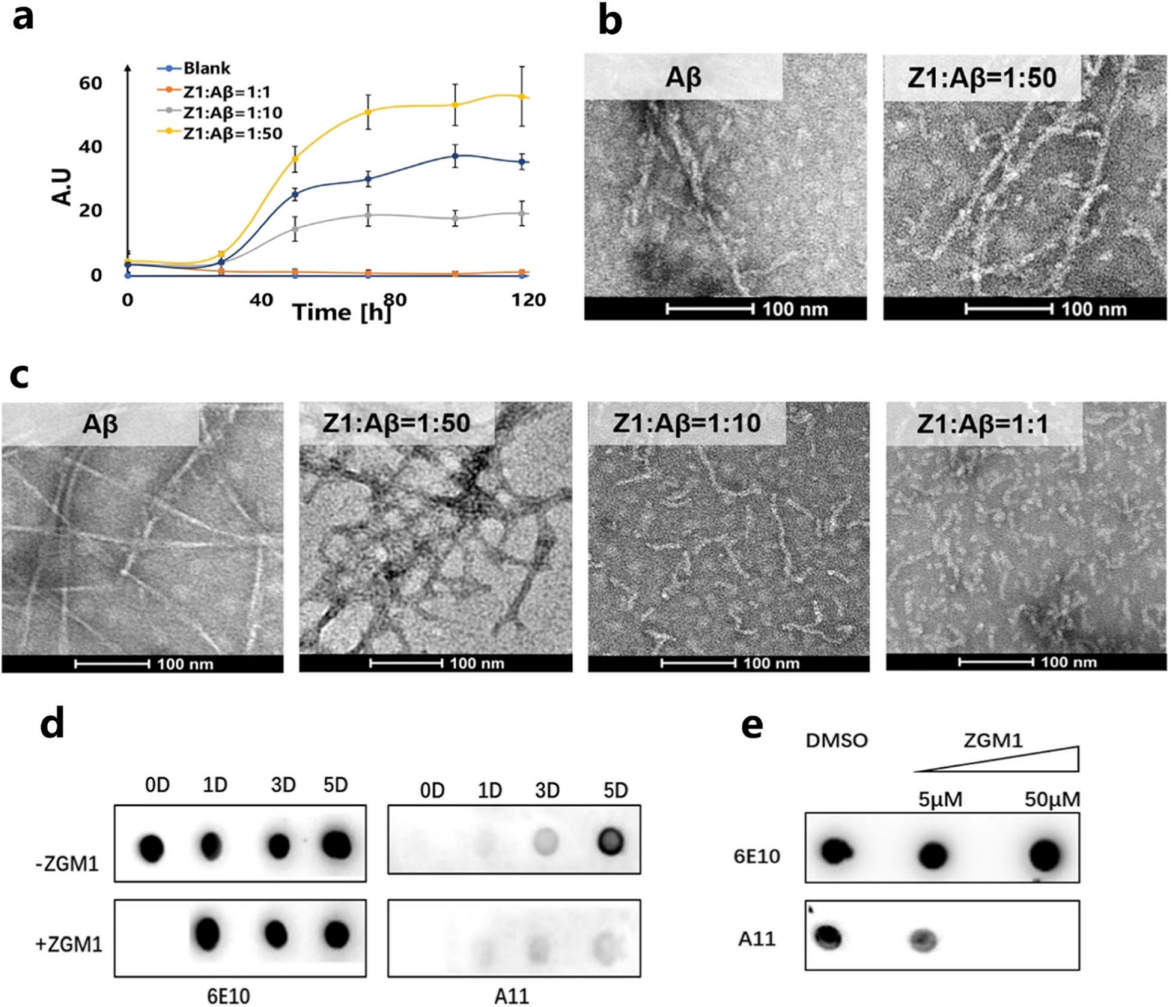
Different concentrations of ZGM1 have different effects on Aβ aggregation. **a** Aβ42 aggregation monitored by thioflavin T (ThT) fluorescence. Aβ (10 μM) was incubated with 20 μM ThT and the corresponding ratio of ZGM1 at 37 °C. The error bars represent the S.D. **b**, **c** Analysis of Aβ42 (50 μM) aggregates by TEM. Samples were incubated with the corresponding ratio of ZGM1 for 24 h (**b**) or for 7 days (**c**) at 37 °C. Scale bars, 100 nm. **d**, **e** Dot blotting of total Aβ (anti-Aβ: 6E10, also recognizes APP) and Aβ oligomers (anti-amyloidogenic protein oligomers: A11). **d** Aβ42 was incubated without or with ZGM1 for 0, 1, 3, or 5 days at 37 °C. **e** Aβ42 was incubated without or with ZGM1 (5 μM, 50 μM) for 5 days at 37 °C

To test whether ZGM1 promoted the conversion of Aβ42 monomers into amyloid fibrils or protofibrils, we mixed 10 μM Aβ42 monomer with ZGM1 at different concentration gradients and incubated the mixture at 37 °C. The content of the amyloid fibrils was monitored by the ThT fluorescence, since Hironobu Naiki and H. LeVine III demonstrated that thioflavin-T (ThT), as a potent fluorescent marker of amyloid proteins, binds fibrils [17, 18]. The results showed that different concentrations of ZGM1 had different effects on Aβ amyloid fibril formation. When the concentration of ZGM1 was the same as the initial concentration of the Aβ42 monomers, we could hardly detect any fluorescent signal, which meant that the Aβ42 monomers were almost completely unable to form any fibril structure during incubation (Fig. 2a). When the signal ratio of ZGM1 and Aβ was 1:10, the signal was weaker than that in the control group (Fig. 2a). Interestingly, the signal significantly increased when the concentration ratio was 1:50 (Fig. 2a).

### Different concentrations of ZGM1 promote the formation of different forms of Aβ aggregates

The diversity in the ZGM1-generated amyloid fibrils indicated that the presence of ZGM1 may lead to the formation of different types of aggregates. To further explore this, we incubated Aβ42 with different concentrations of ZGM1 and observed the morphology of the ZGM1-generated aggregates by transmission electron microscopy (TEM). After incubation for 24 h at a ratio of 1:50 (ZGM1 to Aβ), the morphology of the Aβ aggregates was significantly different from that of the control. More protofibrils and early amyloid fibrils were produced, which showed more branches (Fig. 2b). This phenomenon revealed that ZGM1 may increase the number of seeds during Aβ42 aggregation and explained why the ThT fluorescence signal increased rapidly after 24 h (Fig. 2a). In the absence of ZGM1, the monomers incubated for 7 days aggregated into long and unbranched amyloid fibrils (Fig. 2c). As the concentration of ZGM1 increased, the morphology of the Aβ42 aggregates gradually changed into various forms (Fig. 2c). However, when the molar ratio of ZGM1 to Aβ was 1:50, a network structure was formed (Fig. 2c), which was consistent with the increase in fibrils observed in the ThT assay (Fig. 2a) and the increased presence of bifurcated fibrils at 24 h (Fig. 2b). Interestingly, when the molar ratio was 1:10, Aβ could only aggregate into short fibrils. Furthermore, when the molar ratio was 1:1, no fibrous structure could be observed, and only globulomers could be formed (Fig. 2c), which is in line with the results of ThT assays that showed that no fibril signal could be observed. This phenomenon was similar to off-pathway aggregation [19].

### ZGM1 reduces the production of toxic Aβ oligomers

Studies have shown that soluble oligomers with severe toxicity are intermediates in the on-pathway aggregation process that forms amyloid fibrils and rarely exist in the off-pathway aggregation process [20]. The results of ThT assays and TEM indicated that ZGM1 may accelerate off-pathway aggregation, so it is likely to reduce the production of soluble toxic Aβ42 oligomers during incubation. To validate this hypothesis, we mixed 50 μM of Aβ42 monomers with ZGM1 at different concentration gradients and incubated the mixture at 37 °C. We obtained 2 μl of each sample every other day from day 0 to day 5, and then we spotted these samples onto the nitrocellulose membrane. The samples were subjected to dot blotting (DB) using the 6E10 and A11 antibodies. The 6E10 antibody was used to detect all Aβ42 species, while A11 was used to detect soluble Aβ42 oligomers. The results showed that ZGM1 dose-dependently inhibited the conversion of Aβ42 monomers into Aβ42 oligomers during incubation (Fig. 2d, e), regardless of the type of aggregation it promoted.

### ZGM1 has low cytotoxicity in both SH-SY5Y cells and primary neurons

**Fig. 3:**
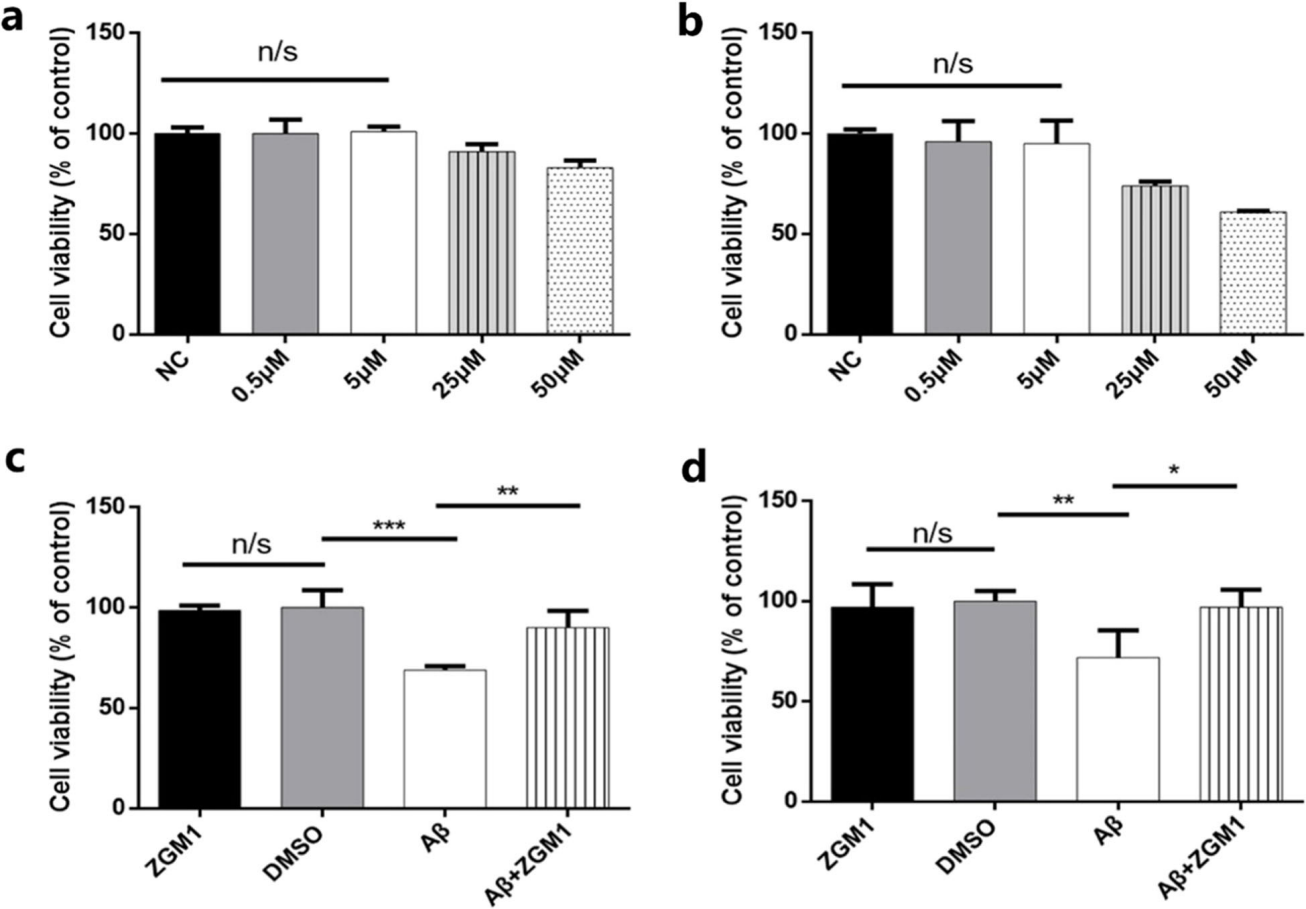
| ZGM1 has low cytotoxicity and can effectively reduce the cytotoxicity of Aβ. **a**, **b** CCK8 assays. SH-SY5Y cells and neurons were treated with different concentrations of ZGM1 for 48 h. Cell viability was normalized to that of the DMSO-treated cells. **a** SH-SY5Y cells. **b** Neurons. **c**, **d** CCK8 assays. SH-SY5Y cells and neurons were treated with ZGM1 (5 μM), Aβ (5 μM), ZGM1+ Aβ (5 μM + 5 μM), and the same content of DMSO as the other samples for 2 days. **c** SH-SY5Y cells. **d** Neurons. The error bars represent the S.D. One-way analysis of variance was performed (*P < 0.05, **P < 0.01, ***P < 0.001; n/s were not significant)

To test whether ZGM1 is cytotoxic, we cultured SH-SY5Y cells and primary neurons from mice in 96-well plates and then treated them with different concentrations of ZGM1 for 48 h. The viability of each group of cells was detected by a CCK8 assay. The survival rates of both SH-SY5Y cells and primary neurons at the effective concentration of ZGM1 were not significantly different from that of the control group containing the same amount of DMSO (Fig. 3a, b). This result indicates that ZGM1 at the working concentration has no significant cytotoxicity in cells derived from the nervous system cultured in vitro.

### ZGM1 reduces the cytotoxicity caused by Aβ in vitro

To test the protective effect of ZGM1 against Aβ cytotoxicity, we cultured SH-SY5Y cells and primary cortical neurons and then treated them with 5 μM Aβ in the presence or absence of ZGM1 for 2 days. The viability of each group of cells was detected by a CCK-8 assay. The survival rate of both kinds of cells treated with Aβ was significantly lower than that of the control group (Fig. 3c, d). After adding 5 μM ZGM1 into the medium, the survival rates of SH-SY5Y and primary neurons were significantly increased (Fig. 3c, d).

### ZGM1 intragastric administration has no significant toxicity toward mice and penetrates the blood–brain barrier

**Fig. 4:**
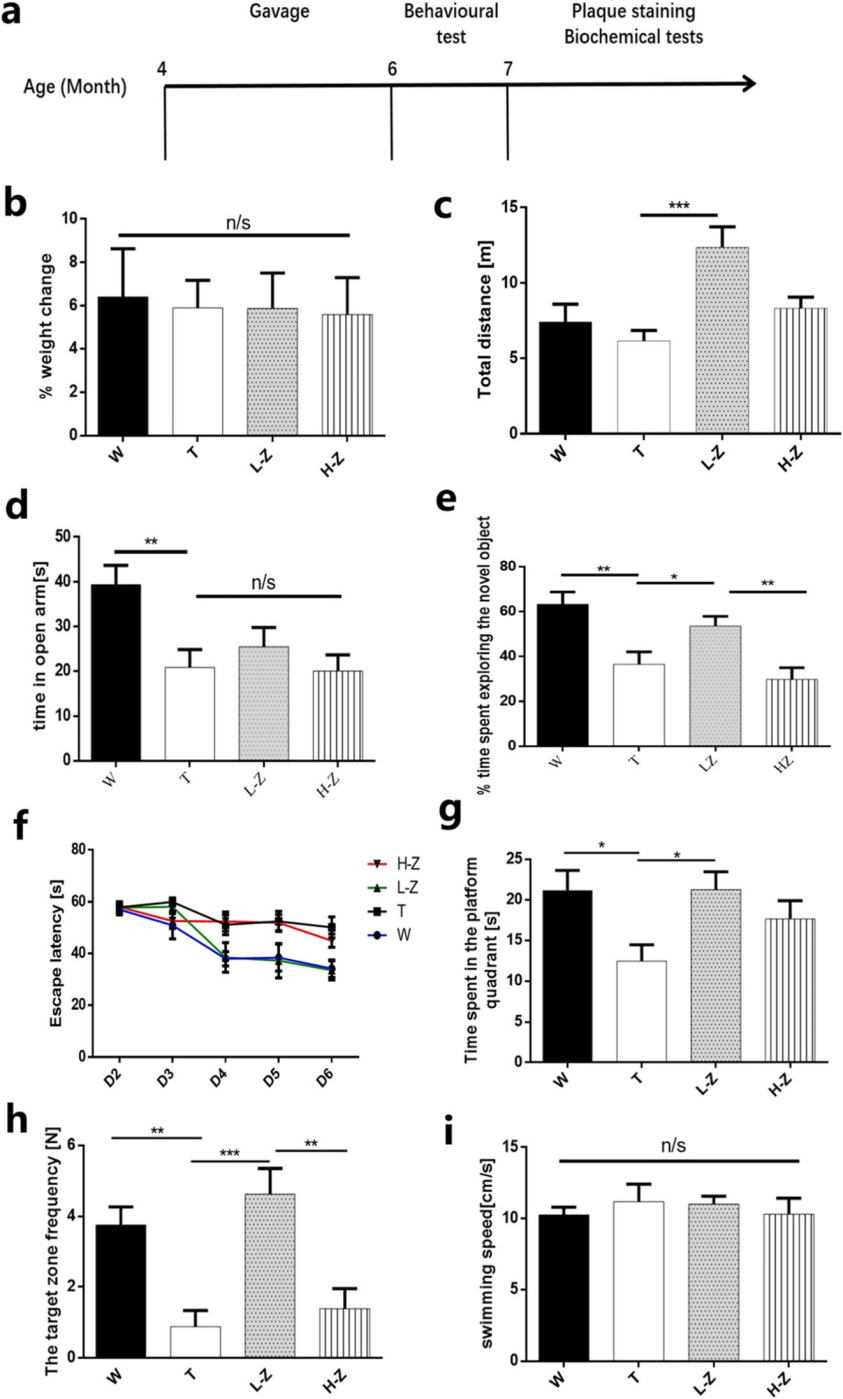
| ZGM1 improves the learning and memory ability of APP/PS1 mice. **a** Time course of the behavioral tests. ZGM1 at 0 (T, male, *n* = 12), 40 (LD, male, *n* = 10) or 120 mg/kg per day (HD, male, *n* = 10) was given to 4-month-old APP/PS1 mice for 2 months by gavage, and their behavioral changes were compared with those of age-matched WT mice (W, male, *n* = 10). **b** % weight change before and after intragastric administration. **c** Total movement distance in the open field for the W, T, LZ, and HZ groups. *P* = 0.00072. **d** Time spent in the closed arms of an elevated plus maze. *P* = 0.0025. **e** % time spent in exploring new objects in the novel object recognition test; from left to right: *P* = 0.0040, 0.033, 0.0044. **f** Hidden platform test in the Morris water maze (for significance, see Additional file 1: Table S2). **g** Time spent in the platform quadrant in the probe test, *P* = 0.022, 0.014. **h** The target zone frequency in the probe test, *p* = 0.0031, 0.00061, 0.0057. **i** Swim speeds in the probe test. The error bars represent the S.E.M. One-way analysis of variance was performed (*P < 0.05, **P < 0.01, ***P < 0.001; n/s were not significant)

Since ZGM1 can prevent the formation of toxic soluble oligomers of Aβ42 and reduce their cytotoxicity in vitro, we further tested whether ZGM1 has a similar effect in vivo. We administered a ZGM1 suspension (0, 40, or 120 mg kg^− 1^ per day in 0.1% sodium carboxymethyl cellulose) to APPswe/PS1-dE9 mice (TG, 4 months old, male; Fig. 4a) by gavage for 8 weeks. During gavage, the mice showed no obvious toxic reactions, such as hair loss or diarrhea, and no mice died due to its administration. The body weight of each group was measured before and after intragastric administration, and the rate of change was calculated. The results showed that there was no significant difference between the groups (Fig. 4b), *P* > 0.7, indicating that ZGM1 had no significant effect on the growth and development of mice.

Mass spectrometry analysis of the mouse tissue after ZGM1 administration (TG, 4 months old, male; 40, 120, or 250 mg kg^− 1^ ZGM1) revealed that ZGM1 can penetrate the blood-brain barrier and become enriched in the brain (Additional file 1: Figure S2a). Two hours after administration, the abundance of ZGM1 in the brain reached a peak concentration of 308 nM (40 mg kg^− 1^), 795 nM (120 mg kg^− 1^), or 1319 nM (250 mg kg^− 1^), and the concentration remained relatively stable until 6 h after administration (Additional file 1: Figure S2b). The drug concentration in the plasma was too low to be detected (40 or 120 mg kg^− 1^).

### TG mice treated with low concentrations of ZGM1 showed hyperactivity in the open field test

The APP/PS1 model mice produced elevated levels of human Aβ because of the expression of mutant human APPswe and PSEN1dE9 proteins. This model is known to have an AD-like phenotype starting at 5 months of age [21]. Behavioral tests were started after the end of ZGM1 administration (6 months old). First, we tested the autonomous exploration behavior of mice through the open field test. The results of one-way ANOVA showed that after administering low concentrations of ZGM1 (40 mg kg^− 1^ per day), the total distance the mice traveled in the open field test was significantly higher than that of the other groups at the same age (*P* < 0.0010 (Fig. 4c), indicating that administering low concentrations of ZGM1 may enhance the curiosity of mice in new areas.

### ZGM1 showed no significant effect on the anxious behaviors of TG mice in the elevated plus maze test

Mental behavior abnormalities such as anxiety and depression are often accompanied with memory and cognitive dysfunction in the pathogenesis of Alzheimer’s disease [22]. We tested the effect of the drugs on anxiety in AD model mice by using the elevated plus maze. The results of one-way ANOVA showed that administering ZGM1 did not increase the time the AD model mice spent in the open arm (*P* > 0.25) (Fig. 4d), while there was a significant difference between the AD model and the WT in terms of this index (*P* < 0.01) (Fig. 4d), which indicates that ZGM1 had no significant effect on the anxiety level of the AD model mice.

### TG mice treated with a low dose of ZGM1 showed memory improvements in new object recognition and the Morris water maze tests

Decreased learning and memory ability, along with cognitive dysfunction, are the most important clinical symptoms of AD patients, especially the decline in short-term memory ability, which is often regarded as the earliest symptom [23, 24]. The new object recognition test operates according to the principle that animals innately like to explore new objects and exploits this to detect the learning and memory ability of animals. This method allows mice to perform learning and memory tests under free and active conditions, which can more closely simulate human learning and memory behaviors and is suitable for the detection of short-term memory ability in mice [25]. The results of one-way ANOVA showed that the percentage of time spent in exploring new objects by the AD model mice was significantly increased after administration of a low dose of ZGM1, and the difference was significant compared to that in mice without ZGM1 administration (Fig. 4e). The percentage of time spent in exploring new objects by mice given a high dose of ZGM1 was not significantly different from that of untreated mice (Fig. 4e). This suggests that low concentrations of ZGM1 can improve the short-term memory of AD model mice, thereby enhancing their interest in new objects in the vicinity.

Finally, to detect changes in the spatial learning and memory ability of TG mice after ZGM1 administration, we conducted Morris water maze tests. In the Morris water maze test, there was no significant difference in the swimming speed of the mice in the administration group and untreated TG mice (Fig. 4i), which indicated that ZGM1 had no significant effect on the swimming ability of the mice. TG mice administered low concentrations of ZGM1 showed significant cognitive improvement (Fig. 4f). However, no significant cognitive improvement was observed in the high-concentration ZGM1 administration group (Fig. 4f). Subsequent spatial probe tests also showed similar results. Regardless of the time spent in the target quadrant or the number of times the mice crossed the target quadrant, TG mice receiving a low dose of ZGM1 were showed significantly better results than untreated TG mice (Fig. 4g, h). However, there was no significant difference between the high-concentration ZGM1 administration group and the untreated TG mice (Fig. 4g, h). These results indicated that intragastric administration of the proper amount of ZGM1 to young TG mice alleviates the symptoms of early cognitive impairment. The administration of a low concentration of ZGM1 had a preventive effect on the occurrence of AD symptoms in TG mice.

However, the Western blot results showed that there was no significant difference in the levels of learning- and memory-related biomarkers in the mouse brain homogenates between groups (Additional file 1: Figure S3), such as AMPA receptor subunit 2 (GluR2). This may be because APP/PS1 mice were still in the early stages of the disease when they were sacrificed, and synapse-associated proteins were only phosphorylated and internalized (Sumasri at al., 2016).

### ZGM1 increases the number of Aβ plaques in APP/PS1 mice

**Fig. 5:**
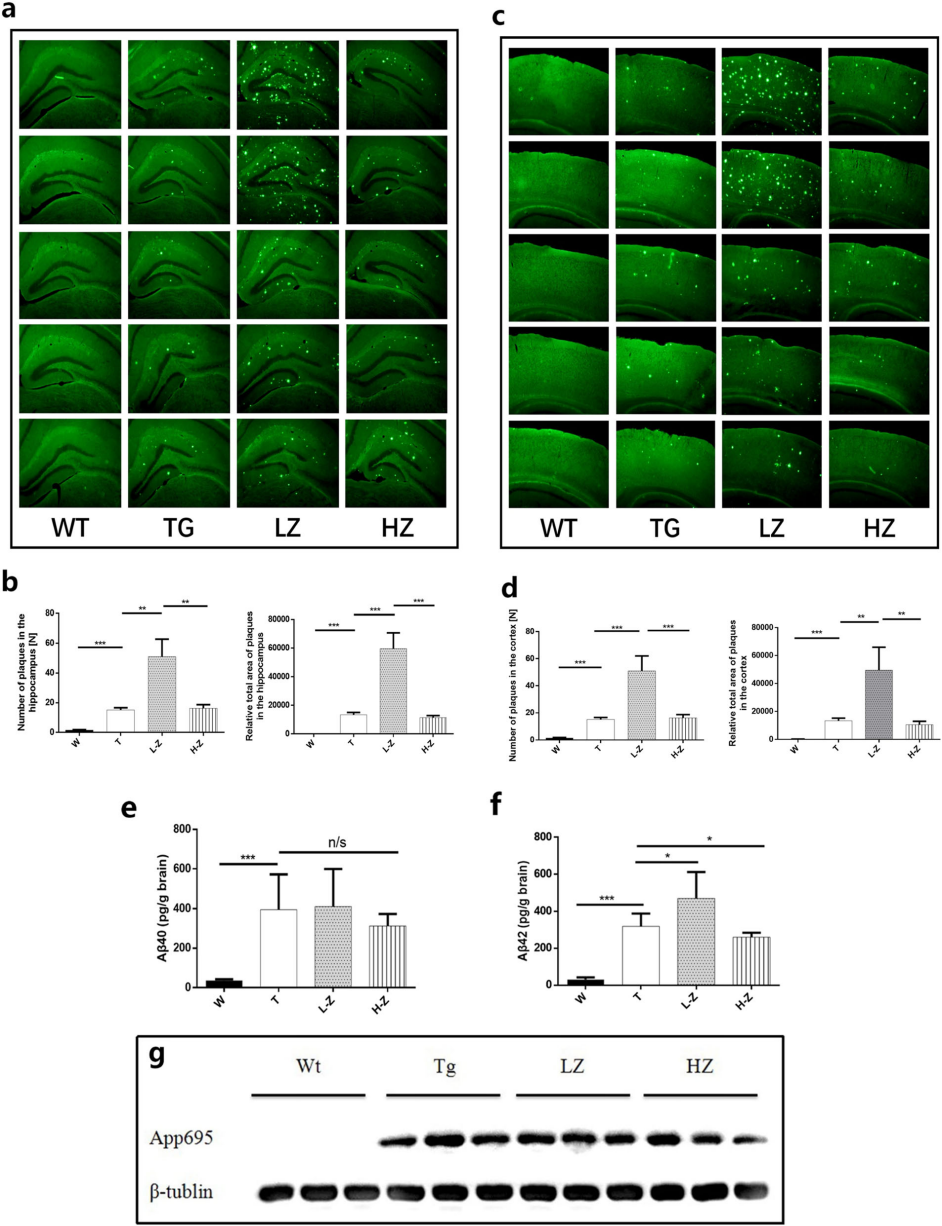
ZGM1 increases the number of Aβ plaques and Aβ42 content in APP/PS1 mice. APP/PS1 and WT mice subjected to behavioral tests were also subjected to brain analyses (*n* = 10~12). ZGM1 at 0 (TG (TG), male), 40 (TG (LZ), male), or 120 mg kg^− 1^ per day (TG (HZ), male) was given to 4-month-old APP/PS1 for 2 months, and their brains were compared with age-matched WT brains (WT(W), male). **a**, **c** ThS-stained Aβ plaques in the hippocampal or cerebral cortex region (scale bars, 500 mm) of each group. Each column of pictures shows the plaque levels of different individuals within the same group to show the individual differences. **b**, **d** Number or relative total area in the hippocampal or cerebral cortex region. **b** Hippocampal region. Number from left to right: *P* = 3.87E−07, 0.0040, 0.0087. Relative total area from left to right: *P* = 1.05E−07, 0.00022, 0.00044. **d** Cerebral cortex region number from left to right: *P* = 1.09E−10, 0.00039, 0.00061. Relative total area from left to right: *P* = 3.00E−09, 0.0071, 0.0042. **e**, **f** ELISA of Aβ40 (**e**) (*P* = 1.17E−05) and Aβ42 (**f**) in total brain lysates (*P* = 1.07E−08, 0.021, 0.027). **g** Western blotting analyses of APP expression in total brain lysates (detected at ~ 100 kDa by 6E10 antibody). The error bars represent the S.E.M. One-way analysis of variance was performed (*P < 0.05, **P < 0.01, ***P < 0.001; n/s were not significant)

After behavioral testing, we sacrificed the APP/PS1 and WT mice. To test the effect of ZGM1 administration on Aβ plaques in the brain, brain slices from each group were stained with thioflavine S (ThS) to observe Aβ plaques in the hippocampus and cortical tissue. Compared with WT mice, a smaller number of plaques appeared in the brains of TG mice (Fig. 5a, c). In contrast, the number of Aβ plaques in the brain tissue of TG mice administered a low dose of ZGM1 were significantly elevated (Fig. 5b, d). The relative area of Aβ plaques in the cortex and hippocampus of TG mice administered a high dose of ZGM1 was reduced, although the difference was not significant. The results of brain slice ThS staining demonstrated that low-dose ZGM1 can promote Aβ aggregation in vivo as well.

In addition, the results of ELISA showed that compared to that of TG (319 ng/g protein), the content of Aβ42 increased by 47% after the administration of a low dose of ZGM1, although the content of Aβ40 in the brain tissue of mice was not significantly changed (Fig. 5e, f). Western blot results showed no significant difference in the APP bands between groups (Fig. 5 g), indicating that the increase in Aβ content was not due to the upregulation of APP expression. We believe that the increase in Aβ42 that we observed in the low-dose group was due to the binding of ZGM1 to Aβ42 to promote its aggregation into amorphous aggregates, which increased the solubility of Aβ42 compared to that observed in the fibrous, aggregated state.

Clinical data have shown that the number of Aβ plaques in the brain is not directly related to the severity of cognitive impairment in patients [2, 26]. Therefore, improvement in learning and memory in the L-Z group was not inconsistent with an increase in plaques. The effect of ZGM1 on Aβ plaques in mouse brains is consistent with the results of the TEM and ThT assays (Fig. 2a–c), which indicated that the effect of ZGM1 on Aβ aggregation in vivo is likely to be similar to that in vitro.

## Discussion

Studies have mainly focused on finding small molecule compounds to inhibit Aβ aggregation or to promote the depolymerization of amyloid fibrils. Many compounds have been found to have both effects. For example, the Wanker group reported that epigallocatechin gallate (EGCG) can inhibit the formation of Aβ fibrils when incubated with monomers [27]. When EGCG was incubated together with preformed Aβ fibrils, Aβ aggregates could be remodeled [28]. Kim et al. found that 4-(2-hydroxyethyl)-1-piperazinepropanesulfonic acid (EPPS) can depolymerize amyloid plaques in the brains of 10.5-month-old AD model mice, thereby improving animal learning and memory function [29]. Such studies have been conducted in vitro and at the cellular level, and the results of animal experiments suggest that these compounds may be more suitable for populations that have developed large numbers of amyloid plaques. Furthermore, another potential risk of this treatment is the production of a large number of Aβ oligomers with strong cytotoxicity during the process of inhibiting aggregation and/or promoting depolymerization. Moreover, compounds that inhibit aggregation/promote depolymerization have a poor effect in early prevention and treatment.

In addition, 2,8-bis-(2,4-dihydroxy-phenyl)-7-hydroxy-phenoxazin-3-one (O4) was reported to promote the aggregation of Aβ monomers and the formation of fibrils [30]. Since studies have shown that AβOs have stronger neurotoxicity than mature fibrils or amyloid plaques, we hope to reduce the toxicity of AβOs by accelerating the aggregation of Aβ monomers or converting them into nontoxic forms, thereby improving cognitive impairment in AD patients or mice.

**Fig. 6:**
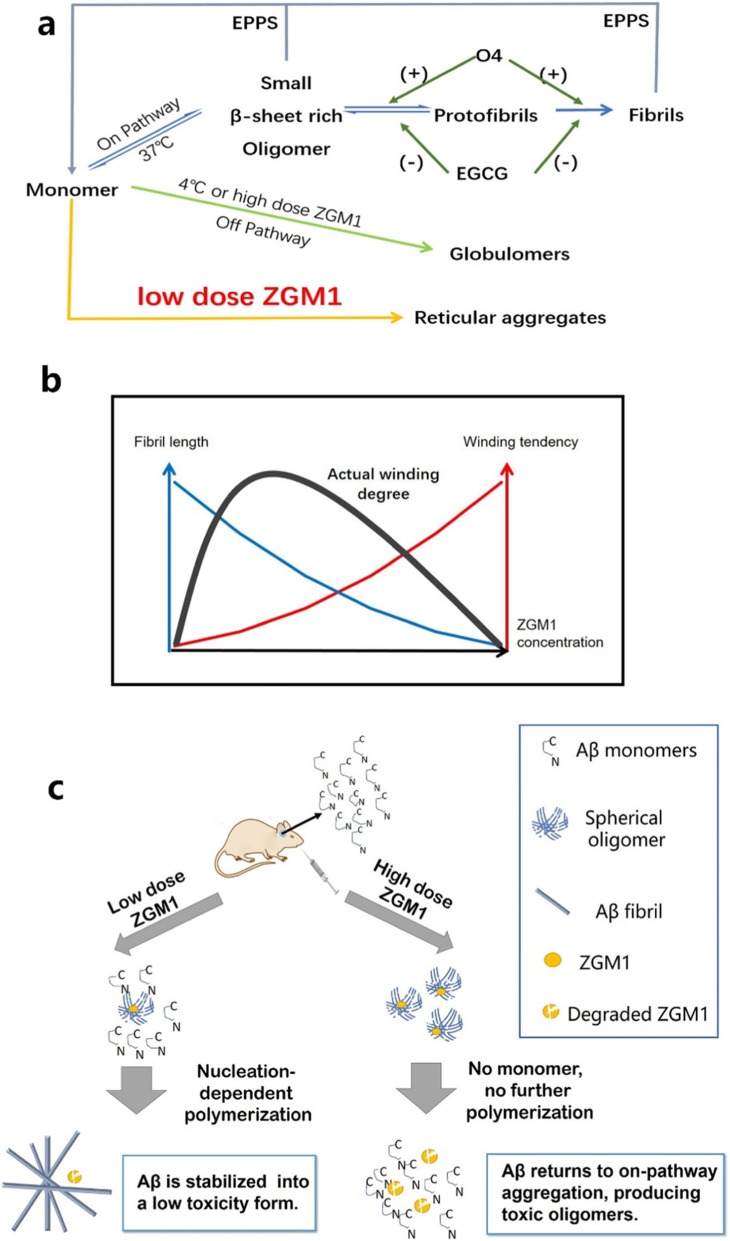
| ZGM1 stabilizes non-β-sheet oligomers at 37 °C and affects the length and entanglement of Aβ amyloid fibrils. **a** Three pathways of Aβ aggregation. Low doses of ZGM1 mediate Aβ aggregation into sponge-like aggregates in a novel pathway and reduce the neurotoxicity of Aβ. **b** As the concentration of ZGM1 is increased, the lengths of Aβ amyloid fibrils are shortened, but they become entangled more easily. When the concentration of ZGM1 was 1% of that of Aβ42, the actual degree of entanglement of the Aβ amyloid fibrils was the largest. **c** Working model of the effects of different concentrations of ZGM1 on Aβ42 polymerization in vivo. A low concentration of ZGM1 promotes the aggregation of Aβ monomers into minimally toxic amyloid fibrils, improving learning cognitive impairment in APP/PS1 mice

Here, we report that [1] a small molecule, the flavanol compound ZGM1, binds to the Aβ42 monomer and switches the Aβ42 aggregation process from the on-pathway to the off-pathway process at 37 °C, reducing the production of toxic AβOs (Fig. 6a) [2]. Low-dose ZGM1 significantly rescued cognitive impairment in APP/PS1 mice [3]. Low-dose ZGM1 administration resulted in a significant increase in Aβ plaque deposits in APP/PS1 mice [4]. During the course of the experiment, ZGM1 showed a beneficial effect on AD at both the cellular and individual levels, and no significant toxicity was observed.

However, the high-dose group did not show better results (Fig. 4b–h). We speculate that this may be due to the different aggregation patterns of Aβ when incubated with different concentrations of ZGM1. The process of Aβ aggregation involves nucleation-dependent polymerization. Besides β-sheet-rich oligomers, it was reported that non-β-sheet globulomers were obtained by incubating Aβ monomers with detergent in buffer [19]. Globulomers cannot be assembled into Aβ fibrils directly and are therefore referred to as off-pathway aggregates. In contrast, oligomers with β-sheet structures are called on-pathway aggregates. Ali Reza et al. have shown that globulomer toxicity is similar to that of Aβ fibrils and is much less than that of β-sheet-rich oligomers, which can be recognized by A11 [31]. Notably, Ahmed et al. obtained globulomers under low temperature and low salt conditions and found that they can be rapidly converted to on-pathway aggregate products such as protofibrils and fibrils at 37 °C [32].

Based on the above studies, we speculated that ZGM1 can mediate the aggregation of Aβ42 monomers into globulomers, which can be used as seeds in fibril formation. The number of amyloid fibrils can be elevated by increasing the ZGM1 concentration. Amplification of the ThT fluorescence signal (Fig. 2a) and the formation of multistart fibrils (Fig. 2b) after incubation for 24 h at a molar ratio of 1:50 (ZGM1 to Aβ) can be considered to be evidence of this. The morphology of the final Aβ aggregates is likely to be affected by both the number and lengths of the fibrils (Fig. 6b). In addition, the total amount of Aβ was constant in vitro, so an increase in the number would produce a decrease in the fibril length. At a low ZGM1 concentration, globulomers promoted the rapid aggregation of the remaining Aβ monomers into long-length fibrils, which were easily entangled with each other (Fig. 2c). When the concentration of ZGM1 was too high, most of the Aβ monomers only aggregated into globulomers (Fig. 2c). Previous research showed that both fibrils and globulomers had low cytotoxicity [32]. However, we suspected that globulomers could return to the on-pathway process and rapidly be converted into toxic oligomers after ZGM1 was metabolized in vivo (Fig. 6c) based on the findings of Ahmed et al [32]. Hence, the level of cognitive impairment in TG mice given high doses of ZGM1 was similar to that in mice without ZGM1 intervention (Fig. 4).

Although the specific metabolic processes and kinetic parameters of ZGM1 remain to be further elucidated, the available results indicated that it may be feasible to accelerate the rapid aggregation of Aβ during the toxic oligomer phase and delay the progression of AD. The rate of Aβ aggregation can be greatly accelerated by a small number of seeds, so ZGM1 can function at concentrations well below the EC50 value. The results of ThT have also demonstrated that ZGM1 can significantly accelerate Aβ aggregation at a concentration of 200 nM. Since mass spectrometry can only detect the abundances of unmetabolized and unmodified ZGM1 prototypes in tissues, it is also possible that other forms of metabolites of ZGM1 may play a role in the pharmacological processes. We also observed that ZGM1 increased the number of Aβ plaques in the brains of mice, which suggested that ZGM1 promoted the formation of amorphous Aβ aggregates. Although these Aβ aggregates are likely to be of low toxicity, the long-term safety of the use of ZGM1 for the early prevention of AD still needs to be further evaluated.

## Conclusion

Currently, drug development for the treatment of Alzheimer’s disease has encountered serious difficulties. The failure of drugs such as BACE and γ-secretase inhibitors, immunotherapeutics against Aβ, and inhibitors of Aβ aggregation suggests that we need to try new strategies. After analyzing the current status of AD drug development, our proposed solution is to accelerate the aggregation of Aβ to reduce the amount of cytotoxic Aβ oligomers in brain tissue. This strategy differs from the existing idea of reducing the total Aβ content and the number of amyloid plaques. The main goal is to reduce the amount of cytotoxic Aβ oligomers in brain tissue. In this study, we tested a small molecule compound (ZGM1) to determine its ability to promote the aggregation of Aβ monomers, mediate a new Aβ assembly process and reduce the amount of Aβ oligomers. Animal experiments showed that ZGM1 can significantly improve cognitive dysfunction in AD model mice, and we found that Aβ plaque deposition in the brain tissue of the mice in the drug-administered group was significantly increased.

## Supplementary information

**Additional file 1:** **Figure S1.** Two-dimensional 1H,15 N correlation spectrum obtained for ZGM1. (HSQC, 1H NMR at 500 Hz, 13C NMR at 125 Hz in CDCl3). Figure S2. ZGM1 concentration in mouse tissue over time after administration. (a) ZGM1 concentration in blood plasma or brain homogenate after administration at a dose of 250 mg/kg. (b) ZGM1 concentration in brain homogenate after administration at a dose of 40 mg/kg, 120 mg/kg and 250 mg/kg. Figure S3. Western blotting analyses of learning- and memory-related proteins in mouse brains. Table S1. NMR data of the ZGM series. Table S2. Escape latency and significance (*p* value) of the hidden platform test in the Morris water maze.

## Data Availability

The data generated during this study are included in this article and the Additional files. All raw data used and/or analyzed during the current study are available from the corresponding author on reasonable request.

## Abbreviations

AD: Alzheimer’s disease
Aβ: β-amyloid
APP: Amyloid precursor protein
AβOs: Aβ-soluble oligomers
ADDLs: Aβ-derived diffusible ligands
ZGM1: 2-(2,3-Dihydroxyphenyl)-5,7-dimethoxychroman-4-one
MST: Microscale thermophoresis
EC50: Half-maximal binding constant
ThT: Thioflavin-T
DB: Dot blot
TEM: Transmission electron microscope
ThS: Thioflavine S
WT: Wild-type mice
TG: APPswe/PS1-dE9 transgenic mice
