# Supplementary material for: Rescue of cognitive deficits in APP/PS1 mice by accelerating the aggregation of β-amyloid peptide
Source: Alzheimers Res Ther. 2019 Dec 17;11:106. doi: 10.1186/s13195-019-0560-6 (PMC6918606; doi:10.1186/s13195-019-0560-6)
Supplement: Supplementary file 1 — Additional file 1: Figure S1. Two-dimensional 1H,15 N correlation spectrum obtained for ZGM1. (HSQC, 1H NMR at 500 Hz, 13C NMR at 125 Hz in CDCl3). Figure S2. ZGM1 concentration in mouse tissue over time after administration. (a) ZGM1 concentration in blood plasma or brain homogenate after administration at a dose of 250 mg/kg. (b) ZGM1 concentration in brain homogenate after administration at a dose of 40 mg/kg, 120 mg/kg and 250 mg/kg. Figure S3. Western blotting analyses of learning- and memory-related proteins in mouse brains. Table S1. NMR data of the ZGM series. Table S2. Escape latency and significance (p value) of the hidden platform test in the Morris water maze. [file 13195_2019_560_MOESM1_ESM.docx]

**Supplementary information**

**Figure S1.** Two-dimensional 1H,15N correlation spectrum obtained for ZGM1

(HSQC, 1H NMR at 500 Hz, 13C NMR at 125 Hz in CDCl3).

**
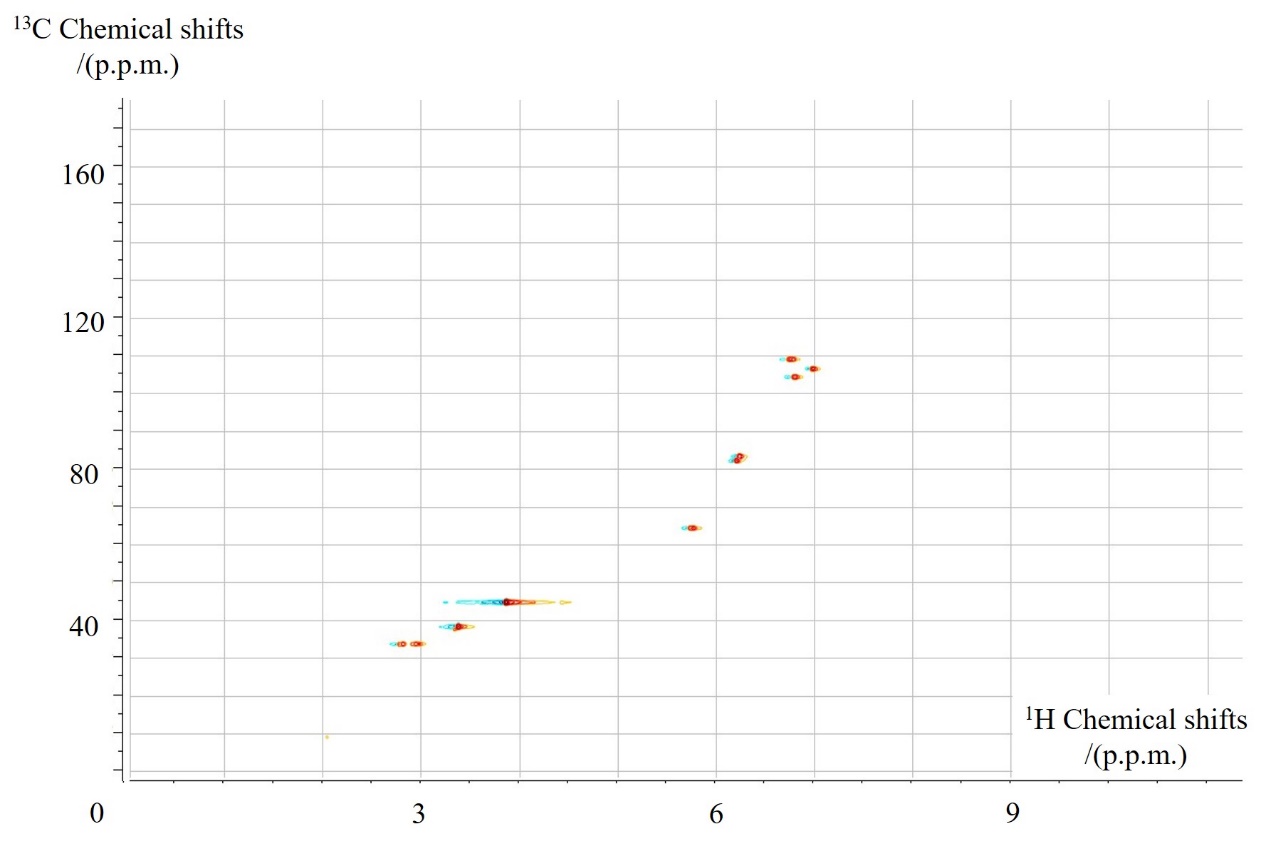
**

**Figure S2 |** ZGM1 concentration in mouse tissue over time after administration. (a) ZGM1 concentration in blood plasma or brain homogenate after administration at a dose of 250 mg/kg. (b) ZGM1 concentration in brain homogenate after administration at a dose of 40 mg/kg, 120 mg/kg and 250 mg/kg.

**a**


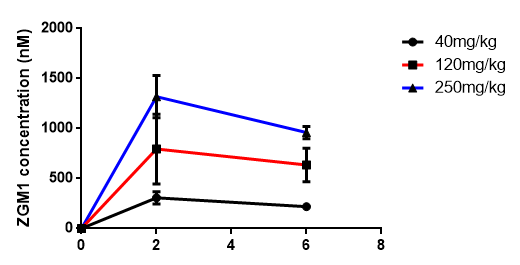


**b**

**Figure S3.** Western blotting analyses of learning- and memory-related proteins in mouse brains.


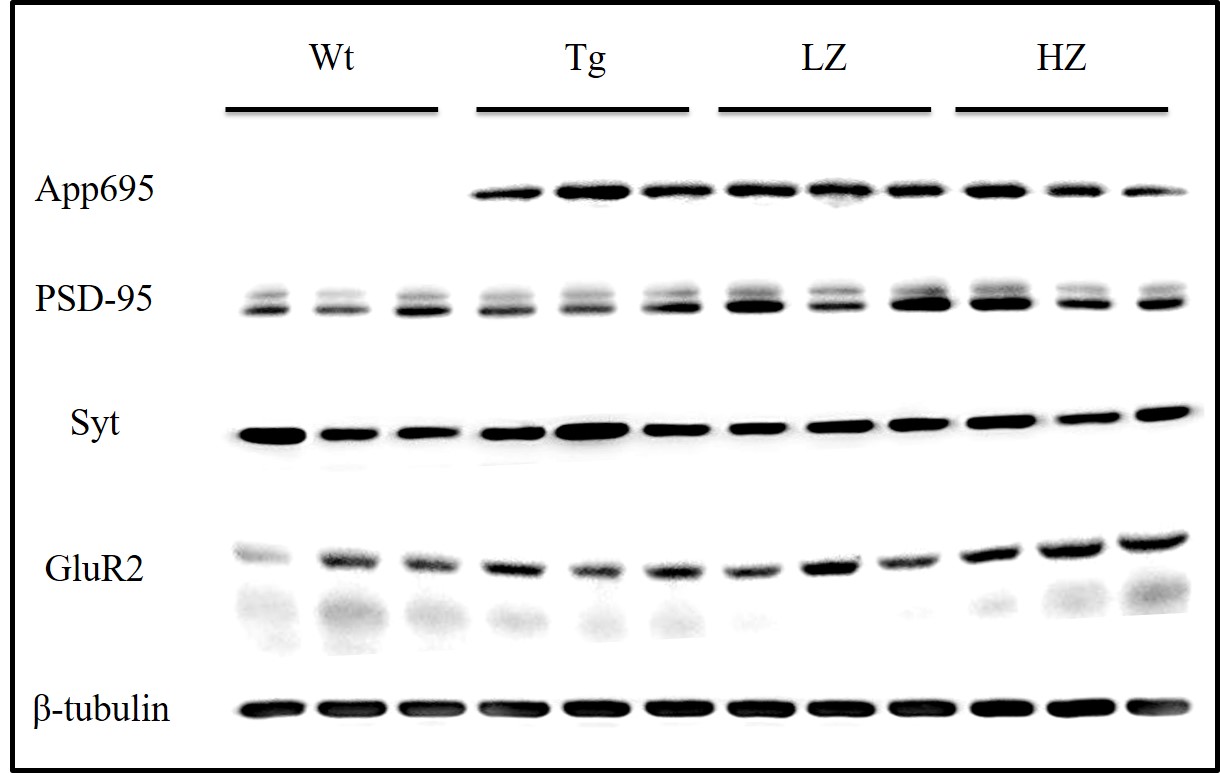


**Table S1.** NMR data of the ZGM series


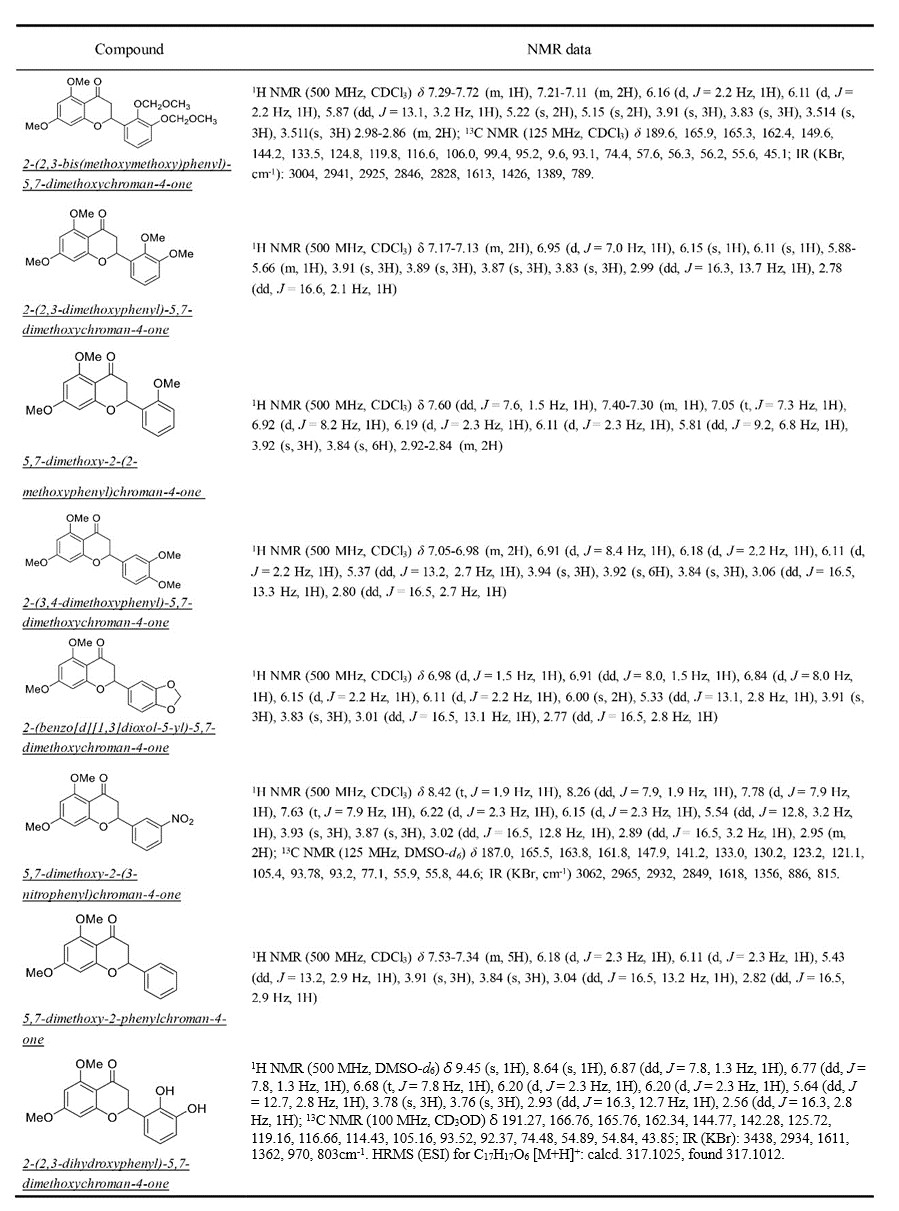


**Table S2.** Escape latency and significance (P value) of the hidden platform test in the Morris water maze.

| **Results from statistical analysis** |
| --- |
| Two-way repeated measures ANOVA |
| Genotype effect: F(1,40) =21.888, P = 0.000 |
| Low-dose ZGM1 effect: F(2,40) = 14.853, P = 0.000 |
| High-dose ZGM1 effect: F(3,40) = 1.264, P = 0.265 |
| One-way ANOVA |
| Day 4: WT(−) vs. TG(−), P = 0.018 |
| TG(−) vs. TG(+), P = 0.088 |
| TG(+) vs. TG(++), P = 0.060 |
| WT(−) vs. TG(++), P = 0.010 |
| Day 5: WT(−) vs. TG(−), P = 0.063 |
| TG(−) vs. TG(+), P = 0.068 |
| TG(+) vs. TG(++), P = 0.065 |
| WT(−) vs. TG(++), P = 0.057 |
| Day 6: WT(−) vs. TG(−), P = 0.005 |
| TG(−) vs. TG(+), P = 0.006 |
| Other comparisons were not significant. |
